# The Role of Psychological Capital in Human Service Professionals’ Work Experiences

**DOI:** 10.3390/ejihpe11030046

**Published:** 2021-06-27

**Authors:** Ilaria Di Maggio, Maria Cristina Ginevra, Laura Nota

**Affiliations:** Department of Philosophy, Sociology, Pedagogy and Applied Psychology, University of Padua, 35122 Padova, Italy; mariacristina.ginevra@unipd.it (M.C.G.); laura.nota@unipd.it (L.N.)

**Keywords:** psychological capital, work experiences, teachers, healthcare professionals

## Abstract

The study was set up as a first exploration of the predictive role of human service professionals’ (i.e., teachers and healthcare professionals) psychological capital (PC) in their perception of work experiences and some core aspects of their own work, such as their efficacy to instill positive resources in their clients, the positive representation of their work and of the results that they can obtain, and positive beliefs about their career growth. Three hundred and eight Northern Italian human service professionals were involved, of which 163 were elementary school teachers of inclusive classrooms and 145 were healthcare professionals in day and residential centers. The regression analyses which were carried out—controlling for age, gender, years of work experience and the typology of the human service jobs—confirmed the predictive role of PC in the efficacy to instill positive resources in one’s clients, the positive representation of the work and of the results that can be obtained, and positive beliefs about career growth. These results have important implications for practice, and they emphasize that specific interventions aimed at promoting human service professionals’ PC may positively impact the effectiveness of their actions for the adaptation and psychosocial development of their clients.

## 1. Introduction

**The Role of Psychological Capital in Human Service Professionals’ Work Experience**

During the 1980s and 1990s, human service jobs such as teachers and healthcare professionals were traditionally considered in studies aimed at analyzing negative outcomes such as burnout, demotivation, fatigue and stress due to the difficult situations they have to face daily, especially concerning their clients’ issues e.g., [1,2]. The research in this field has recently shifted from a paradigm focused on stress and burnout to a paradigm focused on positive outcomes, such as job satisfaction and job engagement [3]. This shift reflects a new trend toward a branch of psychology, i.e., positive psychology, which highlights a set of psychological strengths and optimal functioning, rather than limitations and weaknesses [4].

In this study, we devote our attention to human service professionals who are interested in the inclusion of people with disabilities that work in schools, and in social and healthcare services. As the national and international data show, it is undeniable that they still have to deal with many challenges, especially those related to low pay, career instability, and work–family balance difficulties, as women are primarily engaged in these occupations [5,6]. Specifically, Italian teachers are assisting a growing number of students in their classes, and also have to manage multiethnic classes that increasingly include individuals with vulnerabilities and disabilities [7]. Therefore, teachers have to face extensive difficulties with students, parents and colleagues, related to time pressure, poor working conditions, the need to provide personalized interventions in large classes, and additional administrative responsibilities, such that teaching, especially in inclusive classrooms, has been considered to be one of the most stressful occupations in the last few decades [8].

In day and residential centers, healthcare professionals have to deal with stressors due to heavy workloads, reduced job autonomy, client behavioral and health problems, and long working hours that can facilitate burnout and decrease the effectiveness of caring [9]. Moreover, Italian healthcare professionals work in challenging circumstances due to economic and ethical factors, especially those related to cuts in public spending and the lack of Ethics Consultation Services.

The flourishing of positive psychology prompted several scholars to suggest the necessity of extending the research on workers’ resources (e.g., psychological capital, the perception of support from management and organization, career adaptability), as these resources are useful to cope with working issues [3,10,11] and contribute to wellbeing [12,13,14].

Among these resources, in work contexts, special attention is given to the dimensions of psychological capital—PC [15]. Several studies have found that PC is a validly measureable dimension linked to different workplace outcomes, such as absenteeism, performance and job satisfaction [10,16].

Despite the fact that strengths such as self-efficacy, optimism and resilience are considered to be important for human service professionals to handle stress and burnout [17] no studies have examined PC in relation to some core aspects of their work, i.e., the efficacy to instill positive resources in one’s own clients, the positive representation of work and of the results that can be obtained, and positive beliefs about career growth.

Therefore, the general goal of the current study was to contribute to this issue by investigating the predictive role of human service professionals’ PC—whilst controlling for some sociodemographic variables—on some positive work outcomes, that is, the efficacy to instill positive resources (efficacy, hope, optimism, resilience) in one’s own clients, the positive representation of the work and of the results that can be obtained, and positive beliefs about career growth.

**The Meaning of Psychological Capital**

Although the term ‘psychological capital’ has been addressed in various works on economics and sociology regarding human and social capital [18,19,20], in these study, we focused attention on psychological capital (PC) as defined by positive psychology.

Positive psychology got its start when the psychologist Martin Seligman challenged the field to change from a preoccupation with what is wrong and dysfunctional with people to what is right and good about them. Specifically, positive psychology focuses on strengths rather than weaknesses, and health and vitality rather than illness and pathology [4].

The concept of PC was born within this approach, and—as reported by Luthans et al. [19]—it is beyond human and social capital, and basically consists of “who you are rather than what or who you know” (p.45).

PC is conceptualized as a psychological positive state of development, as opposed to a stable predisposition or trait, characterized by:

(1) having confidence (self-efficacy) to take on and put in the necessary effort to succeed at challenging tasks; (2) making a positive attribution (optimism) about succeeding now and in the future; (3) persevering toward goals and, when necessary, redirecting paths to goals (hope) in order to succeed; and (4) when beset by problems and adversity, sustaining and bouncing back and even beyond (resilience) to attain success [11] p. 3.

These four dimensions are saturated on the second-order core factor of PC. Although, in everyday language, self-efficacy, optimism, hope and resilience are often used as similar and interchangeable terms, several studies [21], [21] have clearly demonstrated that each of these positive resources is theoretically independent, with discriminately valid assessment procedures. In order to assess PC, Luthans et al. [11] developed a specific instrument, the Psychological Capital Questionnaire, which consists of 24 items derived from the Efficacy Scale [22], the Life Orientation Test [23], the Hope Scale [24], and the Resilience Scale [25].

**The Role of PC in Positive Work Outcomes**

As anticipated, several studies highlighted the predictive role of PC in different positive work outcomes. As shown by a meta-analysis carried out by Avey et al. [10] with 51 independent samples of employees, including human service professionals, PC is significantly and strongly related to desirable employee attitudes, such as psychological wellbeing, job satisfaction and organizational commitment, and negatively correlates with undesirable employee work attitudes and behaviors, such as cynicism, frustration, burnout and psychological discomfort. Additionally, Luthans et al. [26] found that a training intervention on PC can improve job performance in workers.

Regarding human service jobs, it was found that PC positively correlates with deep acting and the expression of natural emotion [27], and that it moderates the emotional labor–burnout or job satisfaction relationships [28]. It also positively correlates with work engagement, i.e., vigor, dedication and absorption in healthcare professionals [29].

PC is also related to working behaviors and job performance, suggesting that human service professionals with higher PC are more able to find numerous solutions when problems occur in their jobs, have higher levels of organizational commitment, show less turnover intention, and are more easily adaptive and competent in their job, making them into higher performers then workers with lower PC [10,30]. In addition to the above-mentioned outcomes, particular attention should be paid to some characteristics of the job of the people who work in the field of the inclusion of people with disabilities. These people have to deal with difficult situations every day. Therefore, it is important to focus on the perception of their own work experiences, some core aspects of their own work, and, more specifically, the efficacy to instill positive resources in their clients.

**Efficacy to Instill Positive Resources in One’s Clients**

The current social context is markedly uncertain and unstable, stimulates a negative vision of the future, causes discomfort and discouragement, and reduces progress, improvements in life conditions and personal success. In this context, being able to instill confidence, optimism, hope and resilience in one’s own clients appears to be especially important in order to support people in planning a goal for their future while at the same time facing barriers and difficulties. These tasks may actually turn out to be very hard when these resources are limited [31,32,33].

Instilling positive resources in one’s clients is an important outcome of rehabilitation and treatment interventions [13,14,34,35]. For example, Lewin et al. [13] studying 96 ischemic stroke patients residing in a rehabilitation center, found that instilling in them positive resources such as self-efficacy may help to prevent post-stroke depression. In turn, Wolstenholme et al. [14], involving patients with spinal cord injury, showed that instilling self-efficacy is a crucial factor for benefitting from the hospital experience.

Although no studies have examined the relationship between PC and the efficacy to instill positive resources, Soresi [36] showed that the most hopeful practitioners are more able to restore people’s hope and optimism, maintain their aspirations, and bridge the gap between the events of their present life and the need to look forward into the future. Moreover, Cutcliffe [12] found that healthcare professionals’ hope is one of the factors that contributes to their effectiveness in inspiring and instilling hope in clients who have experienced bereavement and important loss in their life.

On the basis of these findings, we hypothesized the following:

**Hypothesis** **1.** *PC will be positively related to the efficacy to instill positive resources*.

The socioeconomic conditions that have characterized Italy and Europe over the last few years have caused the contexts of health and educational work to become strongly associated with precariousness and instability. Furthermore, these conditions have greatly increased the load and the complexity of the work carried out by human service professionals, and this—together with dealing with particularly complex clients—makes human service professionals more prone to frustration and emotional exhaustion, and exposes them to higher levels of stress [3]. Therefore, having a positive perception of one’s own occupation and of the results that can be obtained is particularly important, as it can be associated with higher levels of satisfaction and work engagement, with a lower inclination to turnover and to leave one’s job, and, above all, with clients’ better levels of health and wellbeing.

As regards the relationship between PC and this dimension, although there are no specific studies [37], involving a sample of Chinese doctors, showed that PC can be considered the most critical dimension in relation to job satisfaction, defined as a global feeling about one’s own job. Additionally, Luthans et al. [11], showed that workers with high PC are more committed to their organizations and more satisfied with their work activities.

Based on the above, we hypothesized the following:

**Hypothesis** **2.** *PC will be positively related to the positive representation of work and of the results that can be obtained.*

**Positive Beliefs about Career Growth**

Career growth can be defined as the individual perception of the occasions of learning and career transitions in one’s own organization and occupation [38]. Holding positive beliefs about the development of their own career and of the chances of the development of their professional category typically produces a significant impact on the efficacy of the interventions that human service professionals implement, and on the levels of satisfaction they get from them [39]. In this regard, Nouri and Parker [39] showed that beliefs about career growth opportunities were related to a stronger commitment to the organization and lower turnover intentions, i.e., two important constructs in human service jobs taking into account that, compared to the general working population, these professionals experience higher levels of psychological distress and burnout [1]. Additionally, Joo et al. [38], found that growth, as one of four factors of work empowerment (i.e., autonomy, feedback, meaningfulness) partially mediated the relationship between PC and work engagement, concluding that employees with high PC are more intrinsically motivated and proactive in work situations, pursue career growth and achievement, and are more engaged with their work.

Thus, we hypothesized the following:

**Hypothesis** **3.** *PC will be positively related to positive beliefs about career growth.*

## 2. Methods

**Participants**

The sample included 308 Northern Italian human service professionals, of which 258 (83.8%) were women and 50 (16.2%) were men. Their mean age was 42.40 years old (SD = 9.53). On average, they had been working for 14.12 years (SD = 10.19), with a range of 1–40 years. Of these participants, 171 (55.5%) had a high school diploma and 137 (44.5%) had a graduate degree. One hundred and sixty-three (52.9%) participants were elementary school teachers of inclusive classrooms, and 145 (47.1%) were healthcare professionals who worked in day and residential centers. The social and healthcare professionals involved in the research hold educational roles aimed at realizing occupational, educational and habilitation interventions.

**Measures**

**Psychological Capital Questionnaire—PCQ** [11].

The PCQ comprises 24 items, and it is composed of four subscales intended to assess hope (6 items, e.g., “Right now I see myself as being pretty successful at work efficacy”), resilience (6 items, e.g., “When I have a setback at work, I have trouble recovering from it, moving on”), optimism (6 items, e.g., “I always look on the bright side of things regarding my job”) and self-efficacy (6 items, e.g., “I feel confident helping to set targets/goals in my work area”). The participants answered each item on a six-point Likert-type scale from 1 (strongly disagree) to 6 (strongly agree). The PCQ demonstrated an adequate confirmatory factor analytic structure across multiple samples [40], and strong internal reliability (α = 0.92; Avey et al., 2009). In this study, hope (α = 0.82), resilience (α = 0.68), optimism (α = 0.60), self-efficacy (α = 0.79) and the overall PCQ (α = 0.86) obtained adequate internal reliability.

**About the Work** [36].

We also used a questionnaire to assess the thoughts that workers have about their work experiences and some core aspects of their work. The instrument includes 34 items, which participants answered on a 6 point scale (1 = It perfectly expresses my point of view and experience; 6 = It does not expresses my point of view and experience). It is composed of three subscales: (1) the efficacy to instill positive resources in one’s own clients (e.g., “With my work I can increase optimism and hope of the people I care”); (2) the positive representation of work and the results that can be obtained (e.g., “The difficulties of people I deal with in my work make me think that there is little that can be done”, a reversed item); (3) positive beliefs about career growth (e.g., “I am sure that my profession will get more recognitions in the future”). Higher scores reveal higher levels of a positive perception of work experiences and the core aspects of the work. The authors reported good construct validity and adequate internal consistency reliability estimates, with alpha values ranging from 0.80 to 0.85 for three factors. The cronbach’s alphas in this study were 0.85 for the efficacy to instill positive resources in one’s own clients, 0.79 for the positive representation of the work and results that can be obtained, and 0.84 for positive beliefs about career growth.

**Procedure**

Day and residential centers and schools were contacted to participate in a project aimed to stimulate human service professionals to reflect on different aspects of their job. The invitation letter contained general information about the project and the aim of the study. The voluntary participants were contacted by a psychologist, who explained, before starting the assessment phase, that the collected information would be protected by professional confidentiality, following the Italian Ethical Principles of Psychologists and the Italian Society for Vocational Guidance (SIO) ethical procedures. Moreover, at the end of the project, all of the participants received a personalized report on their results.

**Data Analysis**

In order to test the hypotheses of the study, preliminary analyses, means, standard deviations and matrix correlations—divided for teachers and healthcare professionals—were carried out [41]. Moreover, an ANOVA and a MANOVA were carried out in order to verify whether any differences associated with the typology of the human service jobs (teachers vs. healthcare professionals) would emerge in the PC and work experiences, and regarding the different aspects of the work (i.e., the efficacy to instill positive resources in one’s own clients, the positive representation of the work and of the results that can be obtained, and positive beliefs about career growth), respectively. Finally, multivariate regression analyses were carried out using SPSS software. Multivariate regression is an extension of a linear regression model with more than one response variable in the model [41].

## 3. Results

**Preliminary Analyses**

The means, standard deviations and correlations for all of the studied variables divided between teachers and healthcare professionals are reported in Table 1. Positive and significant correlations were observed among PC, the efficacy to instill positive resources in one’s own clients, the positive representation of work and of the results that can be obtained, and positive beliefs about career growth. Additionally, the ANOVA on PC revealed a significant effect of the typology of the human service jobs, F(1, 307) = 14.90, *p* < 0.001, η^2^ = 0.046. Specifically, teachers showed higher PC than healthcare professionals. No significant results were found from the MANOVA that was carried out.

**Regression analyses**

In Step 1 of the regression model, the control variables of age, gender, years of work experience, and the typology of the human service jobs were entered. In Step 2, PC was added to the model in order to determine the extent to which it predicted variance in the dependent variables (the efficacy to instill positive resources in one’s own clients, the positive representation of the work and of the results that can be obtained, and positive beliefs about career growth) above and beyond that of the control variables. Specifically, PC added significant unique variance to each of the dependent variables of the efficacy to instill positive resources in one’s own clients (∆*R*^2^ = 0.24, *p* < 0.001), positive beliefs about career growth (∆*R*^2^ = 0.23, *p* < 0.001), and the positive representation of the work and of the results that can be obtained (∆*R*^2^ = 0.22, *p* < 0.001) (see Table 2).

## 4. Discussion

The goal of this study was to analyze the predictive role of human service professionals’ PC on the efficacy to instill positive resources in one’s own clients, the positive representation of the work and of the results that can be obtained, and positive beliefs about career growth.

The results of the regression analyses carried out—controlling for age, gender, years of work experience, and the typology of the human service jobs—confirmed our hypotheses. Specifically, regarding the first hypothesis, it was observed that PC positively predicted the efficacy to instill positive resources in one’s own clients. A possible explanation for this result is that human service professionals with higher PC recognize the importance of these resources for professional and personal development, and also for the management of difficulties arising in school and social settings. Therefore, they believe that their intervention can affect the development of these resources in their clients, and are more greatly motivated to devise activities which are suitable and significant for their growth [13,14].

Regarding the second hypothesis, it was found that PC predicted human service professionals’ positive representation of their work and of the results that they can obtain. An explanatory mechanism for this result is that human service professionals with higher PC believe that they can control their success (efficacy and hope), believe that positive things happen to them at work (optimism), and are more resistant to obstacles (resilience) when compared to those with lower PC. Given the general expectancy of success resulting from optimism and the belief in their abilities resulting from self-efficacy, professionals with higher PC may feel more positive about what they are doing, and may be more likely to be excited about the work outcomes they attain [10].

Regarding the third hypothesis, it was confirmed that PC positively predicted positive beliefs about career growth in human service jobs, suggesting that human service professionals with higher PC are more open to their professional growth and the positive development of their job category. It may be that higher levels of confidence in the ability to succeed in their current job, optimism regarding the future, hope to achieve professional goals, and resilience in facing professional challenges may motivate human resource professionals to take charge of their own professional growth and of the improvement and success of their professional category [10,38].

This study contributes to support the role of PC over and above the control variables of age, gender, years of work experience, and the typology of the human service jobs, in line with Avey et al.’s results [16]. Regarding the typology of the human service job, although teachers showed higher PC than healthcare professionals, the field of work did not add significant variance to the outcomes. This result suggests that PC can actually predict human service professionals’ desirable and positive attitudes, rather than the specific working activities carried out and the difficulties experienced. Thus, PC may entail a greater ability to cope with negative events and more adaptive functioning in a professional field that is generally exposed to high levels of stress and burnout. This could be especially true in the social and economic times we are going through, which increase feelings of pessimism, vulnerability and discomfort both among human service professionals—who more frequently deal with career transitions, precariousness and occupational instability—and among those they take care of professionally [32,33].

The practical implications of this study support the necessity to develop and strengthen human service professionals’ PC. In work contexts, specific interventions could be implemented to foster the four resources of PC, and therefore contribute to human service professionals’ efficacy to instill positive resources in their clients, facilitate the development of a positive representation of their work and of the results they can obtain, and of the positive beliefs about their career growth. This could positively impact the effectiveness of human service professionals’ interventions for the adaptation and psychosocial development of their clients [35,39].

In order to increase PC, as suggested by Luthans et al. [26] individual or small group intervention activities could be implemented. Specifically, at the first stage, a series of specific exercises for each of the four resources of PC could be realized in order to impact their development. Next, more integrative exercises could be proposed in order to integrate the development of the four resources into the understanding and operationalization of the overall construct. Moreover, as suggested by Skovholt and Trotter Mathison [17], starting from specific activities on PC, human service professionals could then be guided in reflection and career counseling activities, to plan the steps needed to improve their work outcomes related to the efficacy to instill positive resources in their clients, to facilitate the development of a positive representation of their work and of the results they can obtain, and to develop positive beliefs about their career growth.

Some potential limitations of the study should be noted. To begin with, even though a regression method was performed to examine “causal” hypotheses, the data collected were cross-sectional and, as a consequence, could not provide evidence of actual causation. In future studies, it would be better to use a longitudinal structural equation method. Moreover, we involved only 308 North Italian human service professionals, who held educational roles aimed at realizing interventions in schools and day and residential centers. Despite the fact that a post hoc G*Power test showed a power of 0.99 for the study, future research should increase the number of participants and should involve human service professionals operating in other contexts, e.g., hospitals and psychiatric centers, etc. Moreover, despite the fact that sociodemographic variables, such as age and gender, and job-related variables, such as the number of years of work experience and the type of human service job, were controlled for, other potential variables such as the educational level were not, and could be considered in further studies. This will aid the generalization of the results carried out. Finally, we obtained low internal consistency for some of the scales; in future research, it would be useful to rely upon multiple methods and informants across the conditions in order to minimize bias due to self-reporting.

## 5. Conclusions

This study provided a contribution to the literature about the role of PC in human service professionals interested in the inclusion of people with disabilities who work at schools and in social and healthcare services. Specifically, it found that PC positively predicted some positive work outcomes, specifically the efficacy to instill positive resources (efficacy, hope, optimism, resilience) in one’s own clients, which can be considered an important outcome of rehabilitation and treatment interventions. Moreover, the study suggested that higher PC in human service professionals can be a resource to develop a positive perception of one’s own occupation and of the results that can be obtained, and about career growth, which contribute to work satisfaction and work engagement.

## Figures and Tables

**Table 1:** Means, standard deviations and correlations.

	Teachers		Healthcare Professionals					
Study Variables	M	DS	M	DS	1	2	3	4
1. Efficacy to instill positive resources in one’s own clients	18.52	3.49	17.82	2.92	-	0.36	0.55	0.50
2. Positive representation of work and results that can be obtained	52.64	6.84	52.09	6.22	**0.30**	-	0.29	0.52
3. Positive beliefs about career growth	24.77	6.02	24.54	5.12	**0.43**	**0.30**	-	0.47
4. PC	110.70	11.96	105.64	11.03	**0.48**	**0.39**	**0.47**	-

Note. The correlations for the healthcare professionals are in bold below the diagonal. All of the correlations are significant at *p* < 0.001.

**Table 2:** Regression analyses with PC, outcomes and covariates.

	Efficacy to Instill Positive Resources in One’s Own Clients		Positive Beliefs about Career Growth		Positive Representation of Work and Results That Can Be Obtained	
Study Variable	Step 1	Step 2	Step 1	Step 2	Step 1	Step 2
Age	0.00	0.02	−0.08	−0.07	−0.03	−0.02
Gender	0.03	0.04	−0.06	−0.05	0.00	0.00
Number of years of work experience	−0.01	−0.05	−0.08	−0.12	−0.09	−0.13
Typology of human service jobs	0.10	0.00	0.09	−0.01	0.08	−0.01
PC		0.50 **		0.49 **		0.48 **
Total *R*^2^	0.01	0.25 **	0.02	0.25 **	0.01	0.23 **
∆ in *R*^2^		0.24 **		0.23 **		0.22 **

** *p* < 0.001.

## Data Availability

Not applicable.
